# AI-driven robotics for optics

**DOI:** 10.1126/sciadv.aee1381

**Published:** 2026-08-19

**Authors:** Shiekh Zia Uddin, Sachin Vaidya, Shrish Choudhary, Zhuo Chen, Raafat K. Salib, Luke Huang, Dirk R. Englund, Marin Soljačić

**Affiliations:** ^1^ Department of Physics, MIT, Cambridge, MA 02139, USA.; ^2^ Research Laboratory of Electronics, MIT, Cambridge, MA 02139, USA.; ^3^ The NSF Institute for Artificial Intelligence and Fundamental Interactions, Cambridge, MA 02139, USA.; ^4^ Department of Electrical Engineering and Computer Science, MIT, Cambridge, MA 02139, USA.; ^5^ Institute for Data, Systems and Society, MIT, Cambridge, MA 02142, USA.

## Abstract

Optical experiments are essential across science and technology, yet their design, assembly, and alignment remain predominantly manual, limiting throughput, reproducibility, and scalability. Automating such experiments is challenging because of stringent precision requirements and the diversity of setups in typical real-world optical laboratory environments. Here, we introduce a platform that integrates generative artificial intelligence, computer vision, and precision robotics to automate free-space optical experiments. The system translates user-defined goals into valid optical configurations, assembles them with submillimeter accuracy, and performs micrometer-scale fine alignment using a robotic tool. It then executes a range of accurate measurements, including beam characterization, polarization mapping, and spectroscopy. This work establishes a platform for reconfigurable optics automation, potentially enabling programmable and adaptive experimental workflows.

## INTRODUCTION

Precise and repeatable control of experimental workflows is a central challenge across the physical sciences. In recent years, the proliferation of data-driven methods and large-scale artificial intelligence (AI) has been transforming how measurements are planned, executed, and interpreted (*1*–*3*). As AI-enabled approaches become more pervasive, the need for integrated automation in bench-scale experiments has grown ever more urgent. Pioneering efforts in many fields have already demonstrated the utility of robotics in accelerating scientific progress. In chemistry, automated platforms now execute multistep syntheses and characterize reaction outcomes with minimal human intervention, enabling rapid exploration of chemical space (*4*–*10*). Similarly, programmable fabrication systems have been harnessed to synthesize materials with targeted properties, perform manipulations with atomically precise control, and perform characterization tasks (*11*–*15*), while cloud-based laboratories have begun offering remote access to state-of-the-art instrumentation at large scales (*16*).

Optics is both a fundamental science, concerned with the study of light and its interactions with matter, and an enabling technology that permeates nearly every scientific domain. It provides the basis for instrumentation in observational astronomy, advanced microscopy techniques in biology, spectroscopic and imaging tools in materials research, and precision experiments in atomic, molecular, and chemical physics. Despite its central role, experimental optics remains predominantly manual in practice and has become a major bottleneck across many disciplines.

Recent work has explored using AI to find or optimize optical configurations in simulations (*17*–*20*) as well as task-specific automation in optical systems, including both machine learning–driven approaches (*21*–*28*) and classical algorithmic or feedback-based control schemes for alignment, calibration, and parameter tuning (*29*–*31*). These efforts demonstrate the feasibility of automating experimental workflows in optics but are typically limited in scope and require substantial human supervision or system-specific engineering. As a result, they do not yet constitute a unified, end-to-end autonomous framework spanning design, implementation, and operation. This inertia stems from major challenges intrinsic to free-space optical systems, which demand reconfigurability, submillimeter precision in alignment, continuous tuning of sensitive optical elements, and in situ diagnostics. Meeting these stringent demands requires an interdisciplinary approach: one that integrates AI, precision robotics, computer vision, engineering, and deep expertise in optical systems. An AI-driven robotic platform for optics automation could overcome these limitations, accelerating experimental workflows. Such a platform would also expand accessibility to complex optical experiments and allow for the exploration of the vast space of optical configurations directly in the laboratory.

**Fig. 1.**
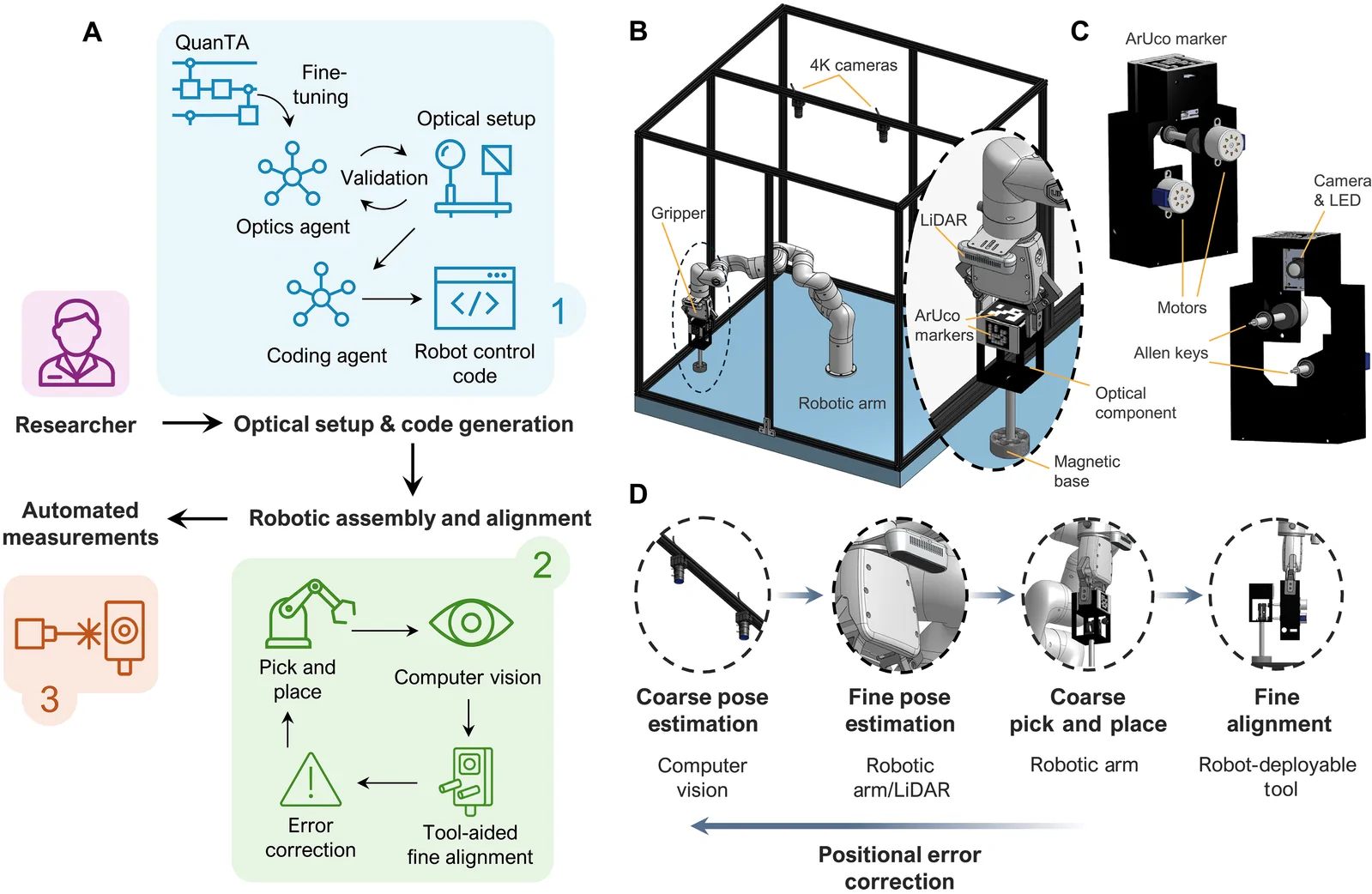
Overview of the AI-driven robotics platform for free-space optics. (**A**) A researcher specifies goals for optics experiments, which are interpreted by an LLM fine-tuned to generate an optical setup design (“Optics agent”). The setup is then validated for accuracy and physical feasibility and translated into robot-executable code for assembly (“Coding agent”). Subsequently, a robotic arm autonomously performs setup assembly, alignment, and optical measurements. (**B**) Schematic of the robotic platform, consisting of a 7-degree-of-freedom robotic arm, computer vision systems, custom 3D-printed casings for optical components, and magnetic bases. (**C**) Schematic of the robot-deployable fine-alignment tool, which consists of two motors with Allen keys mounted on their shafts and an onboard computer vision system enabled by a camera and a white-light LED. (**D**) Pipeline for autonomous assembly of optical setups. First, the computer vision system performs a coarse estimation of the position and orientation of the components in the working area using the 4K cameras. The robotic arm then refines this estimate using the onboard LiDAR sensor. Next, a coarse pick-and-place operation positions and orients the component as desired within the optical setup, achieving submillimeter and subdegree precision. Last, the robot-deployable fine-alignment tool executes adjustments of component degrees of freedom.

Here, we address this critical gap by introducing the first AI-driven robotics platform for free-space optics (Fig. 1) where we aim to automate the three fundamental stages of an optical experiment: (i) design, (ii) physical assembly and fine alignment, and (iii) execution of measurements. At the design stage, we use fine-tuned large language models (LLMs) that interpret user-specified goals and generate valid optical setups within the physical constraints of the laboratory environment. These LLM-generated setups are then translated into robot control code using a second LLM-based agent. A robotic arm equipped with a computer vision system performs pick-and-place operations with submillimeter precision, followed by fine alignment using a purpose-built robot-deployable tool. In the final stage, we demonstrate a range of automated measurements commonly performed in optics laboratories, including beam characterization, polarization mapping, transmission spectroscopy, and real-space setup optimization.

## RESULTS

### Optical setup design using LLMs

Designing free-space optical experiments requires translating high-level experimental intent into concrete spatial configurations of optical components. This requires reasoning about geometric and practical laboratory constraints and laser propagation paths. Humans accomplish this by combining domain knowledge of optical physics with spatial intuition—a capability that has remained historically inaccessible to automated systems. A key challenge in this process is that optical setups are highly sensitive to geometric accuracy: Small errors in component position or orientation can lead to beam misalignment and render an otherwise correct design nonfunctional. Generating such geometrically precise configurations is therefore a nontrivial task, even for state-of-the-art LLMs, which must ultimately commit to explicit spatial placements and angles. While this could in principle be addressed through iterative optimization with physics-based simulation, such approaches introduce substantial computational overhead and pipeline complexity. To bridge this gap, we adopt an approach on the basis of fine-tuning of LLMs to generate optical setups, enabling them to produce physically valid and robot-executable experimental designs from natural language descriptions (Fig. 1). Our goal is not unconstrained discovery of arbitrary optical systems but reliable generation of experimentally relevant configurations within a defined component set and laboratory workspace.

To demonstrate the system’s capabilities, we focus on four canonical optical setups of varying geometric complexity: Michelson interferometer, Mach-Zehnder interferometer, Hong-Ou-Mandel interferometer, and a 4f optical imaging system. We define an optical setup as a list of tuples, with each tuple encoding an optical component identifier, *x*-*y* position, and orientation. For simplicity, we assume all components to have a fixed *z*-position (height). From a single reference of each setup, we generate a dataset through geometric data augmentation using similarity transformations (translations, scaling, rotations, and reflections). These synthetic setups are subsequently filtered to ensure physical feasibility: They must lie entirely within the robot-accessible workspace, have no overlapping components, and avoid internal beam paths intersecting the robotic arm’s footprint (see Materials and Methods). This formulation deliberately constrains the problem to structured variations of experimentally relevant topologies, allowing us to isolate and evaluate the core challenge of mapping language to physically valid layouts under real laboratory constraints.

For each setup in the dataset, we generate natural language descriptions using an autocaptioning step. These captions simulate user inputs with varying degrees of specificity, ranging from high-level objectives (e.g., “Give me a Mach-Zehnder interferometer”) to setups with specific requests [e.g., “Give me a Michelson interferometer setup. The incoming laser beam direction makes an angle of 30 degrees with the x-axis and the setup should have a beam splitter positioned at approximately (20 cm, 3 cm)”]. Together, these steps produce a scalable labeled dataset of physically valid, linguistically varied examples from which the model can learn to generate setups without the need for expensive optical simulation.

We then fine-tune a pretrained LLaMA3.1-8B-Instruct model for this task using Quantum-informed Tensor Adaptation (QuanTA) (*32*), a parameter-efficient fine-tuning method (see Materials and Methods). QuanTA uses quantum-inspired tensor networks to modify model weights, enabling efficient high-rank adaptations that can capture complex structural relationships while using substantially fewer trainable parameters than full fine-tuning. Our fine-tuning strategy involves two sequential stages: First, we train the model on our augmented dataset to learn the fundamental principles of generating physically valid optical layouts (structure learning); second, we continue the training with prompts containing specific user constraints/requests to enhance the model’s ability to interpret and adhere to precise experimental requirements (user request understanding).

Once the LLM generates an optical setup, a validation step evaluates the geometric and physical feasibility of the layout (see Materials and Methods). This step is deterministic and independent of the LLM, ensuring that all accepted configurations satisfy explicit physical and workspace constraints. If the configuration fails this validation, it is returned to the model for refinement in a closed-loop correction process. Validated setups are then passed to a secondary LLM-based agent (based on Claude 3.7), which translates the symbolic layout into executable robotic movement commands for physical assembly in the laboratory (see Materials and Methods). In this stage, the agent does not generate arbitrary low-level control code; instead, it composes sequences of predefined robot primitives (i.e., high-level wrapper functions), ensuring that motion consistency and safety requirements are enforced deterministically at the control layer.

**Fig. 2.**
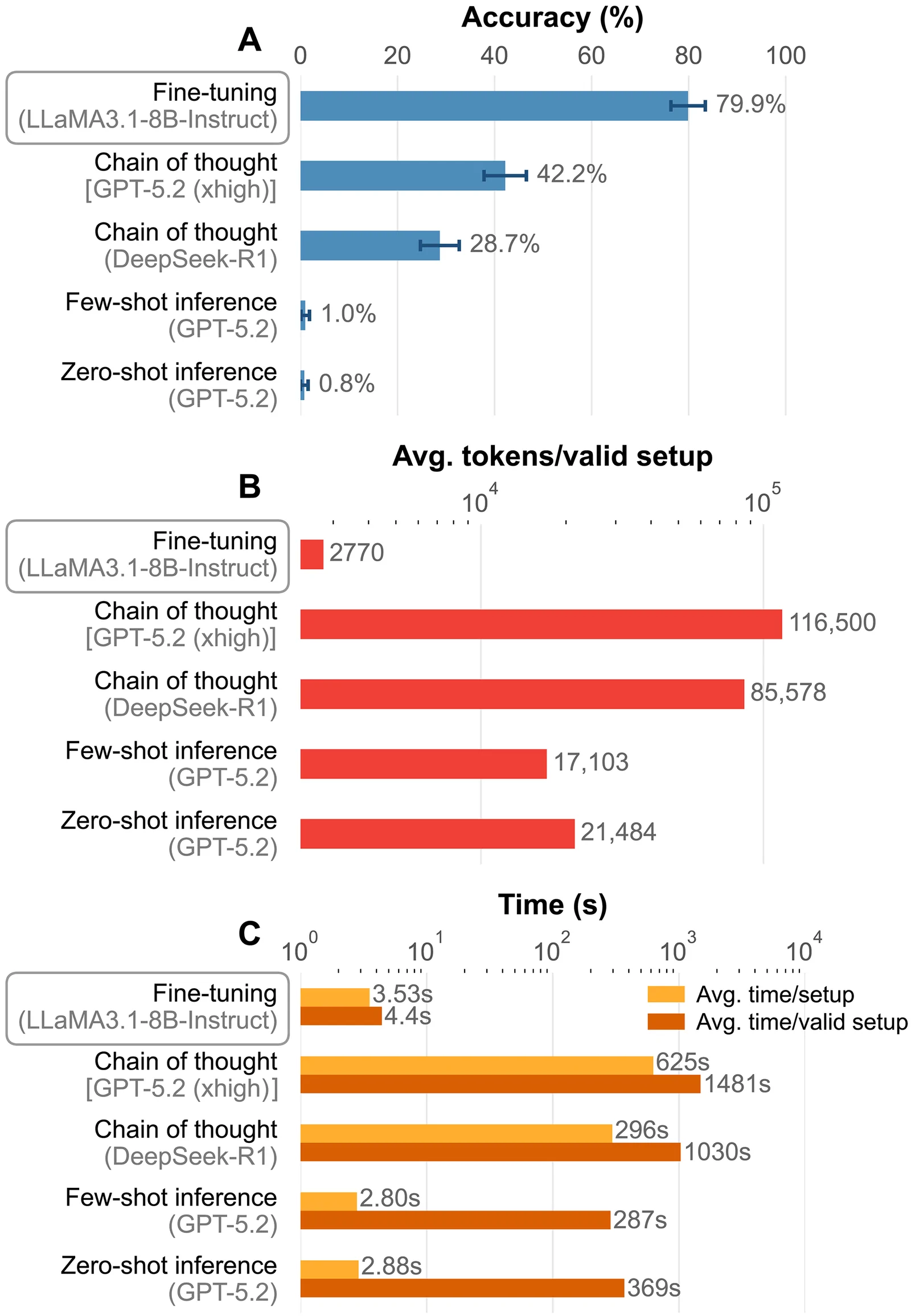
Comparison of prompting strategies for autonomous optical setup generation. (**A**) Accuracy across prompting strategies (with user-specified constraints), averaged over all four setup types. Error bars denote the binomial standard error across evaluations on a test dataset with 128 samples. (**B**) Token usage (log scale) per valid setup generated, averaged over all four setup types, for each prompting strategy. (**C**) Average inference time (log scale) for setup generation for each prompting strategy.

To assess the performance of our fine-tuning approach, we compare QuanTA with three alternative strategies: (i) zero-shot inference using GPT-5.2, (ii) few-shot inference using GPT-5.2, and (iii) prompt engineering combined with chain-of-thought reasoning using DeepSeek-R1 and GPT-5.2 (xhigh) (Fig. 2). We find that our fine-tuned model achieves the highest prevalidation accuracy (∼80%), defined as the fraction of correct setups over total attempts under user-specified constraints, such as exact placement of some components (placement within 1 mm and 0.1° of the requested position), whereas other strategies yield substantially lower accuracies on this task (Fig. 2A). While all approaches would eventually converge to a valid setup after enough iterations through the validation step, the higher accuracy of our fine-tuned model directly translates into reduced computational costs for inference, lower token usage per valid setup, and shorter inference times to obtain a correct solution (Fig. 2, B and C). This highlights the practical advantage of task-specific fine-tuning on small models for optical setup generation, rather than relying solely on repeated inference with general-purpose models.

### Robotic assembly and fine alignment

Following the generation of valid and spatially aware optical setups by the LLM-based design system, the next stage involves the autonomous assembly and fine alignment of these setups using a robotic platform. This system is built around a 7-degree-of-freedom robotic arm (UFACTORY xArm7; see the Supplementary Materials) and is deployed in a standard optics laboratory. The robotic arm is programmed to perform high-precision placement of optical components, including mirrors, lenses, beam splitters, and detectors. We integrate the robotic arm with computer vision, light detection and ranging (LiDAR)–based scanning and depth sensing, and custom-designed optical component holders and magnetic bases, all optimized for robotic manipulation.

Each optical component is placed in a 3D-printed housing that we designed to universally fit standard off-the-shelf optical components. The component is secured to a 3D-printed magnetic base that provides resistance to unwanted motion during assembly and alignment (Fig. 1B). The housing has an ArUco fiducial marker affixed to the top surface that allows for component identification and positioning via stereo 4K visible-light cameras mounted around the workspace. These cameras perform an initial scan of the optical table, allowing the system to estimate component positions and orientations.

**Fig. 3.**
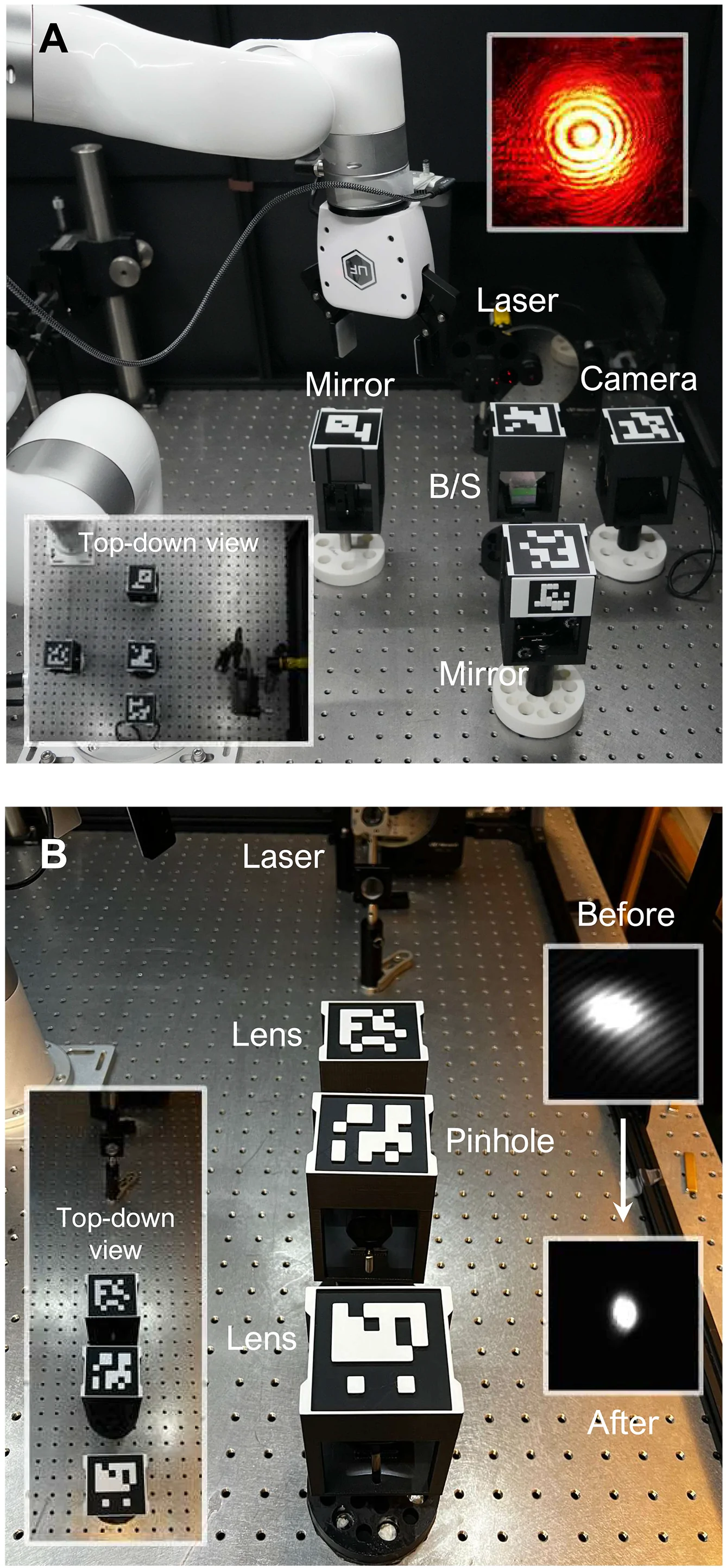
Autonomous assembly and alignment of optical setups. (**A**) Autonomously assembled and aligned Michelson interferometer setup in the laboratory. The insets show a top-down view of the interferometer and a camera image of the achieved interference pattern. (**B**) Autonomously assembled beam cleaning setup (4f optical system) in the laboratory. The insets show the beam profile before and after passing through the setup.

Once a candidate component is identified, the robotic arm approaches it, and a LiDAR sensor mounted on the end-effector performs a high-resolution scan to refine the object’s pose and geometry. This multimodal perception pipeline ensures submillimeter grasping accuracy and minimizes the risk of collision or misplacement. Upon successful pickup, components are placed at their target positions and orientations according to the layout provided either by a user or by the fine-tuned LLM (see movies S1 and S2). During placement, an error correction step ensures positional and angular fidelity by rechecking these parameters using the computer vision system.

For optical setups requiring precision beyond the capabilities of the initial placement, we developed a robot-deployable fine alignment tool (Fig. 1C; also see movie S9). This compact, motorized device is constructed to interface with the fine-adjustment knobs located on standard optical mounts, providing electronically controlled, micrometer-scale adjustments to the component’s degrees of freedom. The tool consists of an Arduino Nano microcontroller, a custom printed circuit board with integrated motor drivers, Allen keys mounted on motor shafts, and a dedicated camera and light-emitting diode (LED) illumination system. For fine alignment tasks, the robotic arm autonomously picks up the fine alignment tool and inserts the Allen keys of the tool into the adjustment knobs of optical components. Alignment adjustments are performed iteratively, guided by feedback from cameras or photodetectors previously placed along the beam path. The complete robotic pipeline is shown schematically in Fig. 1D (see Materials and Methods for more details on the computer vision system, robot pick-and-place operations, custom housing and bases, construction of fine-alignment tool, and correction of positional errors).

To demonstrate the capabilities of the robotic assembly and fine-alignment system, we first ask the AI-driven robotic system to autonomously assemble and align a Michelson interferometer from scratch (Fig. 3A and movies S3 and S4). The optical layout for the interferometer is generated by the fine-tuned LLM in response to a user-specified constraint on the incoming laser beam direction. On the basis of the generated design, the computer vision system identifies the required components—two mirrors, a beam splitter, and a camera—and the robotic arm subsequently places them on the optical table (Fig. 3A). Following initial assembly, the robotic arm deploys the fine-alignment tool to adjust the orientation of the mirrors, ensuring the precise overlap of the two beams of the interferometer. Feedback for the alignment is obtained by blocking each arm and centering the beams in the camera’s field of view. The final interferometer and the resulting stable interference pattern are shown in Fig. 3A.

As a second demonstration, we ask the AI-driven robotic system to autonomously assemble a 4f beam cleaning setup consisting of two lenses and a pinhole. As before, the optical layout is generated by the fine-tuned LLM after specifying the incoming laser direction. The robotic arm places the components to create a 4f setup, and a camera images the beam before and after setup assembly (Fig. 3B; also see movie S10). With the vision-based positional error correction loops, we achieve high reliability for pick-and-place and tool deployment in these tasks; however, autonomous recovery from catastrophic failures remains an important but currently unimplemented feature.

### Automated optical measurements

Following the successful autonomous assembly and fine alignment of optical setups, we proceed to execute automated optical measurements with our platform. While the assembly process involves static placement of components, many optical experiments require dynamic operations such as translating, rotating, or adjusting optical elements in real time. These dynamic measurement tasks demand the same level of precision, robustness, and autonomy as the assembly process but introduce additional challenges related to motion control. Moreover, measurements are also integral to the assembly phase itself—for instance, verifying laser alignment and characterizing beam quality during setup construction. Thus, the integration of real-time optical diagnostics and measurement capabilities is critical to achieving fully autonomous experimentation.

Leveraging the robotic platform’s ability to manipulate optical elements with high spatial and angular accuracy, we demonstrate automated execution of optical measurements. In this section, we show this functionality across five examples of distinct tasks: (i) beam direction and waist tracking, (ii) beam quality characterization through beam matrix measurement, (iii) polarization mapping via spatially resolved Stokes parameter extraction, (iv) angle- and wavelength-resolved spectroscopy of multilayer photonic crystals, and (v) real-space optimization of a beam expansion setup.

**Fig. 4.**
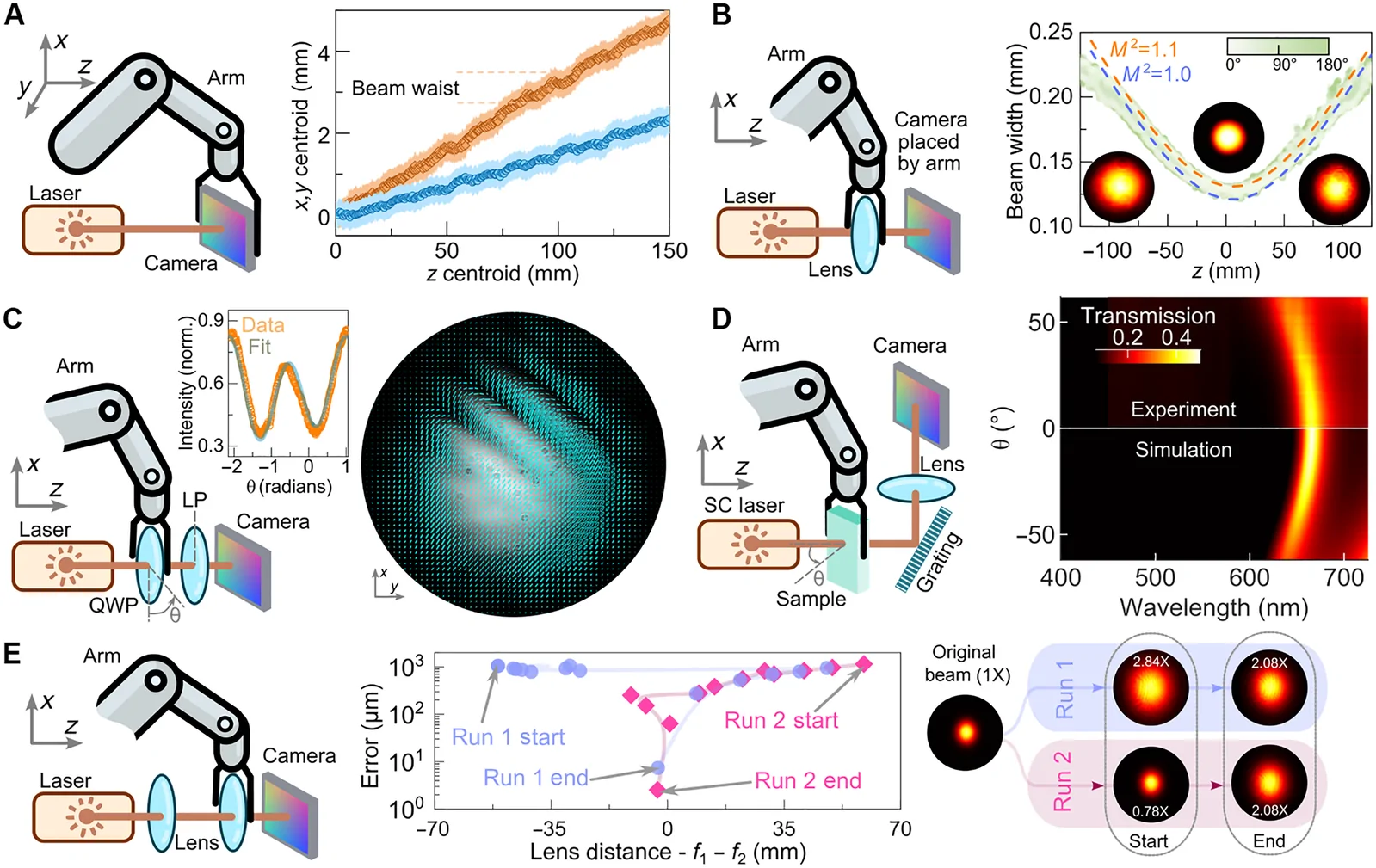
Automated optical measurements, characterization, and optimization tasks. (**A**) The robotic arm moves a camera linearly, tracking the centroid of the laser beam to measure its direction and width. In a typical configuration, beam angles along the *x*, *y*, and *z* axes are measured to be 88.16 ± 0.05°, 89.16 ± 0.05°, and 2.03 ± 0.04°, respectively, averaged over 230 measurements. (**B**) The robotic arm places a lens in the beam path and moves the camera in the *z* direction, capturing images. These data are used to calculate the beam matrix and beam quality factor $M_{\text{eff}}^{2}=1.07\pm 0.03$. (**C**) The robotic arm assembles a setup consisting of a camera and a linear polarizer (LP) in the beam path. The beam passes through a birefringent film (BRF), creating spatial variations in the polarization. The robotic arm holds a QWP and rotates it, while the camera captures images. These images are used to calculate the Stokes parameters and plot polarization ellipses for every point in space, superimposed on the intensity image. (**D**) The robotic arm assembles a setup consisting of a grating and a camera to create a spectrometer and then holds and rotates a multilayer photonic crystal to obtain the angle- and wavelength-resolved transmission spectrum. (**E**) The robotic arm places a $f_{1}=60$-mm focal length convex lens in the beam path and repeatedly places a $f_{2}=125$-mm focal length lens at different locations with the goal of achieving $f_{2}/f_{1}=2.083\times$ beam expansion. Two such runs with different initial positions of the lens are shown; both ultimately converge to the same lens distance. Beams before and after real-space optimization are also shown.

To determine the propagation direction of a collimated laser beam, the robotic arm picks up a camera module and positions it such that the beam is centered within the field of view. The robotic arm then translates the camera while keeping the beam centered in the camera’s field of view. By recording the beam centroid through this motion, the beam’s angle relative to the laboratory axes is calculated using a linear regression fit (see movie S5). Similarly, the beam waist can be directly measured from the camera images (Fig. 4A).

Precise spatial characterization of laser beams is essential in a wide range of experiments, from beam shaping and mode matching to the calibration of complex optical systems. The complete spatial characterization of the beam can be determined via a measurement of the second-order moments of the Wigner distribution, commonly referred to as the beam matrix, which represent the beam’s spatial intensity and phase in both position and momentum (or angle) space. This matrix records beam properties such as width, divergence, astigmatism, and ellipticity. The components of the beam matrix can be measured using cylindrical and spherical lenses and a camera to image the beam at different planes along the propagation axis and at different positions.

To measure the beam matrix, the robotic arm places down a camera and translates a lens in the beam path, capturing the transverse intensity profile at multiple propagation distances (see movie S6). From these images, second-order spatial and angular moments are computed, yielding a full 4×4 beam matrix (Fig. 4B)

$$\begin{array}{c} M=\left(\begin{array}{cccc} \langle x^{2}\rangle & \langle \mathrm{xy}\rangle & \langle x\theta_{x}\rangle & \langle x\theta_{y}\rangle \\ \langle \mathrm{xy}\rangle & \langle y^{2}\rangle & \langle y\theta_{x}\rangle & \langle y\theta_{y}\rangle \\ \langle \theta_{x}x\rangle & \langle \theta_{x}y\rangle & \langle \theta_{x}^{2}\rangle & \langle \theta_{x}\theta_{y}\rangle \\ \langle \theta_{y}x\rangle & \langle \theta_{y}y\rangle & \langle \theta_{x}\theta_{y}\rangle & \langle \theta_{y}^{2}\rangle \end{array}\right)=\\ 10^{-7}\left(\begin{array}{cccc} 0.13\;m^{2} & 0.06\;m^{2} & 0.08\;m & -0.02\;m\\ 0.06\;m^{2} & 0.12\;m^{2} & 0.27\;m & 0.00\;m\\ 0.08\;m & 0.27\;m & 2.80 & 0.04\\ -0.02\;m & 0.00\;m & 0.04 & 3.06 \end{array}\right) \end{array}$$

Here, *x* and *y* are the spatial coordinates of the beam, and $\theta_{x}$ and $\theta_{y}$ are the angular divergences in the *x* and *y* directions, respectively. The diagonal elements describe the beam’s spread (e.g., $\langle x^{2}\rangle$ is the beam width in the *x* direction), while the off-diagonal elements describe correlations between position and angle or between different spatial directions. The effective beam propagation ratio is measured to be $M_{\text{eff}}^{2}=\frac{4\pi }{\lambda }{\left[\text{det}M\right]}^{1/4}=1.07\pm 0.03$, where λ=632.8 nm is the wavelength of light. The estimated uncertainty in this measurement is on the order of a few percent, which is comparable to values typically reported for commercial $M^{2}$ measurement systems (often quoted at ±5%).

We next turn our attention to the characterization of spatially varying polarization fields, which is essential for identifying polarization singularities and vectorial field distributions in beam optics applications. To show this functionality, the robotic arm assembles a setup consisting of a linear polarizer and camera and then inserts and rotates a quarter-wave plate (QWP) along the beam path (see movie S7). The intensity at each camera pixel I(θ) as a function of QWP angle θ follows

$$I\left(\theta \right)=\frac{1}{2}\left(S_{0}+S_{1}\;{\text{cos}}^{2}2\theta +S_{2}\;\text{sin}\;2\theta \;\text{cos}\;2\theta +S_{3}\;\text{sin}\;2\theta \right)$$

By fitting this expression at each pixel, the spatially resolved Stokes parameters $S_{0},S_{1},S_{2},\;\text{and}\;S_{3}$ are extracted. From these, the degree of polarization and the orientation of polarization ellipses are computed and superimposed on the beam intensity image (Fig. 4C).

We next demonstrate automated angle-resolved spectral characterization of multilayer photonic crystals, a task commonly performed to measure the bands of periodic photonic materials. This measurement typically requires precise and repeatable angular sweeps, making it labor-intensive and sensitive to alignment errors when performed manually. The robotic arm first assembles a setup consisting of a diffraction grating and a camera positioned along the path of a supercontinuum laser beam. It then picks up a multilayer photonic crystal [consisting of alternating layers of silicon (Si) and silica (SiO_2_) with an embedded defect layer to form a cavity] housed in a 3D-printed casing. The arm then rotates the photonic crystal through a range of incidence angles (see movie S8). At each angle, the transmission spectrum is recorded, yielding the complete wavelength- and angle-resolved transmission spectrum (Fig. 4D) (see Materials and Methods for details).

To demonstrate the robotic arm’s capability for real-space optimization, we constructed a beam expander consisting of two convex lenses with focal lengths $f_{1}=60$ mm and $f_{2}=125$ mm. While the theoretical optimal separation is $f_{1}+f_{2}$, practical considerations such as lens positioning within housing assemblies and finite lens thickness necessitate experimental optimization to achieve the desired beam expansion ratio of $f_{2}/f_{1}$. To demonstrate this real-space optimization, the robotic arm first positions the first lens. An adaptive gradient descent algorithm then determines the optimal placement of the second lens by iteratively varying its coordinates and minimizing the error function defined as the absolute difference between the target and measured beam widths. Figure 4E shows the optimization error as a function of lens separation for two experimental runs, each initialized from positions displaced to either side of the theoretical optimum. Both optimization sequences converge to the correct lens position roughly within 10 iterations, successfully achieving the target beam expansion ratio of $f_{2}/f_{1}=2.08$, as confirmed by the beam profile measurements shown for the original beam and the beams recorded before and after optimization for each run.

In the Supplementary Materials, we report the time to completion for various steps in these experiments. These five demonstrations collectively highlight the flexibility of robotic systems for executing high-precision optical measurements. Together, they establish that a robotic arm can serve as a versatile experimental tool in optics and photonics, effectively replacing a range of custom, purpose-built characterization instruments with a single reconfigurable platform.

## DISCUSSION

We have presented an AI-driven robotic platform capable of designing, assembling, aligning, and operating free-space optical experiments, demonstrating that full-stack automation in optics is both accurate and technically achievable. By integrating AI, a robotic arm guided by computer vision, and modular custom alignment tools, we have established an automation pipeline that substantially reduces manual intervention and unlocks a new regime of speed, reproducibility, and remote operability in optics.

For LLM-based optical setup generation, we have shown that our fine-tuning approach outperforms conventional prompt engineering–based or zero-shot methods with respect to physical validity, user compliance, and token efficiency. This indicates that domain-specific fine-tuning, combined with structural constraints, can produce models capable of reasoning over physically grounded design spaces—a capability of increasing importance for AI-assisted experimentation across disciplines.

The robotic system addresses a persistent bottleneck in optics: the precise placement and alignment of free-space optical components. Our demonstration of automated optical measurements—including beam characterization, polarization mapping, spectroscopy, and optimization tasks—illustrates the breadth of tasks amenable to full automation. These examples represent generalizable workflows that can be extended to material characterization, imaging applications, and remote beam monitoring, particularly in hazardous environments such as high-power laser and x-ray facilities.

Looking ahead, several opportunities exist to further enhance this platform. A more systematic analysis of end-to-end repeatability across experiments would provide insights into system performance and help identify remaining sources of variability. In addition, extending the platform to more complex three-dimensional (3D) geometries, heterogeneous components, and less structured environments remains an important next step. For setup alignment and continuous optimization under real-world nonideal conditions, a promising direction is the incorporation of reinforcement learning algorithms (*33*, *34*). This is particularly relevant in optics, where the reward signal can be sparse and highly intermittent during alignment, posing a challenge that may be overcome with modern machine learning techniques. Another avenue is the integration of simulation-in-the-loop frameworks, where optical modeling tools inform both LLM-based setup generation and robotic execution, leading to self-optimizing workflows. Last, remote operation and cloud-based optical laboratories will enable distributed and democratized access to advanced optical experimentation.

## MATERIALS AND METHODS

### Optical setup dataset generation

To construct a diverse and laboratory-aware dataset of optical layouts, we developed a data augmentation pipeline centered around a library of canonical optical setups: Michelson interferometer, Mach-Zehnder interferometer, Hong-Ou-Mandel interferometer, and a 4f optical system. Each setup is encoded as a list of component tuples—each specifying a component ID (e.g., mirror and beam splitter), spatial coordinates (*x* and *y* in cm), and optical axis orientation (in degrees). We assumed all components to have a fixed height in the *z* direction. The first tuple with the component ID “000” is always a ray representing the incoming laser beam. The spatial coordinates within this tuple are those of any point lying on the ray, and the orientation component is its angle with the *x* axis.

To expand this initial set and introduce geometric variability, we applied randomly chosen similarity transformations to each reference layout. In particular, for each predefined reference setup, we generated multiple setups via random combinations of scaling, in-plane rotation, translations, and mirroring across the *x* and/or *y* axis. Each transformation was applied to the full component list, updating both spatial coordinates and optical axis orientations accordingly.

Resulting setups were subjected to rigorous physical validation to ensure experimental feasibility. Validation criteria included the following: (i) All components must lie within the reachable circular workspace of a 61-cm radius, (ii) no component may intersect the robot’s 15-cm-radius circular footprint at the origin, (iii) intercomponent spacing must exceed individual component dimensions (7.5 cm by 6.4 cm) to avoid spatial overlap, and (iv) internal beam paths must not intersect the robotic arm’s footprint, determined by constructing a complete graph from the component coordinates and checking for overlap. Setups failing any of these constraints were discarded. The code for optical setup generation and validation is available in (*35*).

### LLM fine-tuning

To enable the translation of natural language experimental goals into precise and structured optical layouts, we fine-tuned the LLaMA3.1-8B-Instruct model using a parameter-efficient fine-tuning strategy. In particular, we used QuanTA (*32*), a method inspired by quantum circuit structures and tensor networks that enables efficient high-rank updates to pretrained model weights. Compared to conventional low-rank adaptation methods, QuanTA offers increased representational capacity with comparable computational efficiency, making it well suited for capturing the complex structural and geometric constraints inherent to optical setup design.

A consistent system prompt was used throughout both the fine-tuning and inference stages to clearly define the task scope, specify the desired output format (structured lists of component tuples: ID, *x*, *y*, and orientation), and impose fundamental constraints such as workspace boundaries and component non-overlap. Full details of the system prompt and dataset specifications are provided in (*36*).

#### Fine-tuning protocol

Our fine-tuning protocol involved two sequential stages.

##### Structure learning

In the first stage, the LLaMA3.1-8B-Instruct model, initialized with the system prompt, was fine-tuned on the optical setup dataset (described above) comprising an equal but random mixture of pairs of autocaptioned natural language descriptors and their corresponding structured optical layouts for the four photonic setups. The primary goal of this stage was to enable the model to learn and internalize the knowledge behind the production of syntactically correct and physically plausible component arrangements, consistent with the basic spatial rules defined during dataset generation.

##### User request understanding

Following the initial structure learning, the model underwent further fine-tuning using prompts representing more specific user requests, such as prescribed component positions or beam path characteristics (e.g., “Describe a compact Michelson interferometer optical system. The system should have a laser beam entering at 318.9 degrees.”). While the target outputs remained structured component lists, the prompts introduced more nuanced constraints, improving the model’s ability to flexibly interpret and satisfy user-defined experimental requirements. This stage is further split into two sessions. In the first session, we train on samples where the user always asks for a fixed number of three requests, allowing the model to understand user requests in a relatively simple format. We relax this condition in the second session to further guide the model in interpreting flexible user requests.

To further improve the diversity of samples, parts of user prompts were generated independently using various templates, with varying precision requirements, language style, notation differences, etc. Together with the randomly generated setup coordinates and angles, this created an exponentially large data distribution, accurately mimicking possible real human inputs and preventing the model from relying on specific patterns in the generated sample pairs [examples of input-output pairs can be found in (*36*)]. The training curves are shown in the Supplementary Materials.

#### Hyperparameters

During fine-tuning, we chose QuanTA dimensions of 64-16-4, resulting in 143.13M training parameters (1.75% of the full model). We used the AdamW optimizer with a weight decay of 0.01 throughout the training. During the first stage and the first session of the second stage, we used a peak learning rate of 0.00001, and during the second session of the second stage, we chose a peak learning rate of 0.000002. For each stage or session, we started the training with an initial linear warm-up, followed by a cosine decay. We used a batch size of 8 throughout the training, and each stage or session was trained for only a single epoch, with no samples seen twice by the model. The full training required ∼8500 graphics processing unit (GPU) hours with A100 GPUs and 80GB VRAM on each GPU.

#### Other prompting strategies

To establish baseline performance benchmarks, we evaluated two contemporary LLMs: GPT-5.2 and DeepSeek-R1. For the GPT-5.2 model, we assessed performance under two distinct prompting conditions: (i) zero-shot prompting, providing the model solely with the user request and the required output format specification, with minimal examples for demonstrating the output format (the same prompt as the one used during fine-tuning of the LLaMA3.1-8B-Instruct model), and (ii) few-shot prompting, augmenting the prompt with illustrative examples of valid pairs of user input and model output. For the DeepSeek-R1 model and GPT-5.2 (xhigh), we used the same few-shot prompt while allowing the model to perform a few-shot chain-of-thought reasoning. This approach was structured to guide the model through step-by-step reasoning for optical layout construction, supported by representative examples.

#### Structure of the prompts

We summarize here the structure of the prompts used in our experiments. Across prompting strategies, we kept the overall task specification fixed and varied, mainly the amount of demonstration data provided to the model. In all cases, the prompt framed the task as optical setup generation under physical and geometric constraints, with the requirement that the output follows a strict machine-readable format. The prompt has five main parts:

1) The first part defines the task and domain. The prompt specifies that the model should generate valid optical arrangements for a constrained laboratory workspace.

2) It also provides a component vocabulary. The prompt lists the allowed optical elements together with standardized three-digit component codes, such as laser, mirror, beam splitter, camera, photodiode, lens, aperture, and polarization elements. This reduces ambiguity in both naming and output representation.

3) It defines a set of canonical templates. The prompt specifies four standard optical systems, namely the Michelson interferometer, Mach-Zehnder interferometer, Hong-Ou-Mandel interferometer, and a 4f optical system, as reference layouts with component identities, positions, and angles. The generated configuration is required to be derived from exactly one of these templates through allowable geometric transformations such as scaling, rotation, or reflection. This converts an open-ended design problem into a constrained transformation problem.

4) The prompt imposes explicit physical and formatting constraints. The prompt specifies workspace limits, robot reach limits, a forbidden inner footprint region, component footprint dimensions for overlap checking, and a strict output format. The desired output is a list of components with coordinates and angles represented in a nested tuple format with fixed units and precision. Making these constraints explicit improves consistency and simplified evaluation.

5) Last, it includes a generation and validation procedure. The prompt instructs the model to identify the requested template, apply only valid transformations, verify workspace constraints, check for overlap, preserve the transformed relative geometry, and output the result only if all checks passed. Thus, the prompt specifies both the target format and the procedure used to construct valid candidate solutions.

The main difference between prompting strategies was the use of demonstrations. In the zero-shot setting, the model received the task definition, constraints, and output schema without worked examples. In the few-shot setting, the same core prompt was augmented with valid and invalid input-output examples spanning different optical systems and common failure modes, including outside robot reach, overlap, invalid transformation, and incorrect component sets. These examples clarified the distinction between valid and invalid outputs and helped enforce the intended constraints. In the fine-tuned setting, the same task structure was retained, but the behavior was learned from supervised examples shown during training rather than inferred only at inference time. The full prompt can be found in (*36*).

### Validation of optical setups and LLM performance

To ensure that optical setups generated by the LLM were accurate and adhered to physical and geometric constraints, we used a validation procedure that extended the checks used during optical dataset generation. First, we applied a set of rule-based spatial constraints to the model-generated setup: All components must lie within the robot’s 61-cm radius reach, remain outside the 15-cm-radius circular footprint of the robot at the origin, and maintain non-overlapping placement on the basis of their 7.5-cm by 6.4-cm physical dimensions, and internal beam paths must not intersect the robotic arm’s footprint.

Passing these spatial checks does not guarantee that the laser beam will correctly propagate through the setup, as the component orientations or positions may still be physically inaccurate. To address this, we performed an additional validation step that verified the structural correctness of the generated setup against a known reference. We did this by attempting to identify a similarity transformation that mapped the LLM-generated setup onto one of the predefined reference setups of the same type. This is framed as a geometric alignment task, where the candidate transformation must preserve component identities and maintain approximate spatial relationships within tolerance bounds. If such a transformation exists, the generated setup is deemed valid because of its equivalence to the corresponding reference configuration. This step ensured that the model had not only obeyed spatial constraints but also produced a layout that structurally corresponds to an accurate optical arrangement of components.

The accuracy of all prompting strategies was measured against a newly generated test dataset with 128 samples for each setup using a generation temperature of 0.5 to moderate output randomness. The test dataset spanned a range of user-request conditions, including different setup size descriptors, optional laser input angle constraints, and component-level requests on the *x* position, *y* position, and orientation. These requests included both exact constraints and approximate language such as “about,” “around,” and “roughly,” and the numerical targets covered a broad portion of the robot workspace and a wide range of component orientations. Representative examples include a 4f system with a lens requested near (20, 20) cm, a Mach-Zehnder interferometer with a beam splitter requested at (7.1, −16.2) cm and a photodiode orientation of 222°7′, and a Michelson interferometer with a beam splitter requested near (25.3, −40.0) cm at 173.3°. The accuracies and user compliance scores are reported in Fig. 2. Full prompt-level evaluation records and validation results for all setups are available in (*36*) under the model-specific JSON files.

### Generation of robot motion code

In this part of our workflow, we used Claude 3.7, an LLM, to transform high-level interferometer design specifications into executable robotic control code. By leveraging predefined wrapper functions that discretize the optical assembly task, we systematically translated the design parameters directly into robotic instructions. The process involved providing Claude 3.7 with a list of tuples with component specifications in the format [component ID, *x* position, *y* position, orientation angle] from the LLM design along with a mapping between component identifiers and their corresponding ArUco markers of the hardware for computer vision recognition. After describing the system’s functional capabilities through four wrapper functions (system initialization, component pickup via visual recognition, precise placement, and robotic arm homing), Claude generated a Python main function that systematically processed the interferometer design. The resulting code initialized the vision and robotic systems, parsed the design data, appropriately filtered nonrobotic components, and implemented an assembly sequence that handled component identification, retrieval, precise placement, and safe arm positioning between operations. This procedure achieved the optical geometry specified by the LLM optical design. The full code base can be found in (*37*).

### Computer vision system

The robotic system’s vision pipeline is built around a stereo vision setup composed of two 4K ELP cameras mounted 23 cm apart. Initial calibration was conducted using a standard checkerboard pattern, with images collected at varying positions on the optical workbench. Intrinsic and extrinsic parameters of the stereo pair were obtained using the OpenCV cv2.calibrateCamera and cv2.stereoCalibrate functions. The calibration outputs were validated by comparing the predicted baseline offset to the physical camera separation, confirming submillimeter discrepancies. These calibration parameters were stored in a serialized NumPy archive (stereo_calibration2.npz) for use in subsequent 3D position estimations during coarse alignment operations.

To enable robust and accurate object detection, we used the ArUco fiducial markers. Each optical component is tagged with a unique identifier drawn from a 6 × 6 binary dictionary comprising more than 800 distinguishable codes. These tags are detected using the cv2.aruco module, which computes the component’s pose via the cv2.solvePnP algorithm using previously derived camera intrinsics. While the *x* and *y* positional estimates from ArUco detection were sufficiently accurate for coarse positioning, the *z* coordinate was instead inferred from a depth measurement. For this, we deployed a robot-mounted Intel RealSense depth camera equipped with LiDAR. After the ArUco tag was centered in the camera field of view, the LiDAR sensor provided a high-accuracy depth estimate, enabling precise *z*-coordinate acquisition. The robot’s end-effector was then translated vertically from a known camera-to-gripper offset to achieve accurate engagement with the object.

The precision and robustness of this system allowed for successful detection and manipulation even with smaller ArUco tags, as demonstrated in the fine alignment tool deployment. To facilitate consistent image acquisition, the workspace was illuminated with diffuse white-light LED strips integrated into the overhead structure. Additional directional LEDs were positioned in the fine-alignment tool to enhance tag visibility. To suppress glare and reduce errors from ambient light sources, light-blocking panels were installed around the frame perimeter.

To support microscale adjustments, we developed an auxiliary vision routine dedicated to centering the fine-alignment tool. An ArUco marker affixed to the housing of the tool is tracked to guide lateral adjustments, aligning the tool precisely with rotational knobs or alignment targets. This system has been validated across a range of component geometries and operates reliably under varied lighting conditions.

A potential limitation of the current implementation is the need for uniquely assigned ArUco tags across all components. In practice, this is mitigated by the large dictionary size and spatial separation of simultaneously visible tags. In multirobot configurations, each robot can use a dedicated camera and ArUco ID space, supporting scalability for distributed collaboration in large or reconfigurable optical assemblies. The code for computer vision is available in (*37*).

### Optical component casing and bases

To facilitate reliable robotic handling of diverse optical components, we designed and fabricated universal housings and bases using fused deposition modeling 3D printing with polylactic acid filament using the Bambu X1E 3D printer. Each housing was tailored to securely enclose optical components while presenting a standardized external geometry. This uniformity eliminates the need for the robotic arm to adapt dynamically to variations in component shapes and sizes, simplifying grasping and manipulation.

An indentation feature was incorporated into each housing, precisely aligned to the robot’s claw gripper dimensions. This design ensures repeatable, stable engagement of the gripper during pickup and transport, greatly reducing the likelihood of slippage or misalignment during handling.

For optical component positioning and identification, ArUco fiducial markers were affixed to the top and side surfaces of each housing. Using these fiducial markers, the system achieves robust visual tracking, even under moderate occlusion or variable lighting conditions.

To ensure stable and repeatable placement of optical components, each unit is supported by a custom-designed base optimized for tabletop experimental environments. The bases were fabricated using fused deposition modeling 3D printing with polylactic acid filament. To prevent sliding and enhance frictional stability, each base was equipped with a rubberized bottom layer. In addition, multiple neodymium magnets were embedded within the lower section of the base. The combined effect of the rubber and magnets greatly increases the frictional force between the base and the optical table, reducing unwanted sliding, tipping, and rotation under typical laboratory vibrations or minor external disturbances (e.g., those caused by the insertion of the fine-alignment tool).

### Robotic pick-and-place operations

The robotic arm executes pick-and-place operations by integrating global and local visual feedback with precise end-effector control. For each task, the system is first provided with the unique identifier corresponding to the desired optical component. The global stereo vision system, composed of two overhead 4K cameras, detects the ArUco markers affixed to the component’s casing and computes its (*x*, *y*) position and orientation relative to the optical table.

Following coarse localization, the robotic arm approaches the component using a predefined trajectory that first elevates the end-effector above the tallest components in the workspace, thereby minimizing collision risks before descending to engage the target. During the final approach phase, a secondary camera mounted on the robot’s end-effector provides high-resolution, close-range visual feedback. This enables fine alignment of the gripper with the standardized indentation designed into each casing, ensuring a secure and precise engagement during pickup.

Upon successful acquisition, the robotic system can perform a variety of manipulation tasks. These include translational movements along prescribed paths, such as following a laser beam propagation line, or rotational adjustments along the polar and azimuthal angles for component alignment. For placement operations, the system receives target (*x*, *y*) coordinates and orientation data. The robot translates the component to the desired location and gently deposits it onto the optical table.

Before finalizing placement, the end-effector camera performs a local verification and correction routine. Minor translational or rotational deviations are adjusted in situ to ensure alignment within the specified tolerance bounds. This dual-stage visual feedback—combining global position estimation with local fine correction—enables highly accurate, repeatable pick-and-place performance, which is critical for the assembly and realignment of complex optical experimental setups.

We note that the low-level motion planning and trajectory execution are handled through the proprietary application programming interface of the robotic arm. For consistency in inverse kinematics solutions, the arm is reset to a predefined home configuration before each operation. While this procedure ensures reliable execution, it limits direct access to trajectory-level metrics (e.g., path length, waypoint count, etc.), and systematic optimization of motion efficiency is beyond the scope of the present work. The code for robotic arm control is available in (*37*).

### Correction of positional errors

Despite the high accuracy of the robotic placement system, occasional positional errors were observed. These inaccuracies arise from two primary sources: variable magnetic attraction between component bases and the optical table and mechanical slippage at the gripper interface resulting from wear of the rubber contact pads.

To mitigate these errors and ensure reliable and robust assembly of setups, we implemented an automated correction protocol on the basis of postplacement verification. After each placement, the computer vision system reevaluates the actual position of the optical element and compares it against the target coordinates. If the distance between the detected and intended positions exceeds a predefined tolerance threshold, the robot initiates a re-placement procedure. For all experiments reported in this work, we set this tolerance threshold to 0.5 mm.

This feedback-correction loop ensures that components are repositioned until they fall within the acceptable positional tolerance threshold. Empirically, most components require only a single placement attempt to meet this threshold. While this process may appear inefficient, the average placement time remains lower than that of a human operator. Even with occasional multiple attempts, the robotic system maintains a net speed advantage, as human placement times typically exceed 20 s per component.

### Fine alignment tool

The fine alignment tool is a compact, robot-deployable device designed to perform precision microadjustments on standard optical mounts (Fig. 1C and movie S9). The tool integrates an Arduino Nano microcontroller, a custom-designed printed circuit board incorporating dual motor controllers, a camera module, and a dedicated illumination system. All components are enclosed in a 3D-printed low-profile housing.

The embedded camera is centrally mounted within the housing of the tool and aligned coaxially with the tool’s mechanical axis. A ring of surface-mounted LEDs is positioned directly above the camera, providing uniform illumination and mitigating shadows from external light sources.

The core actuation mechanism consists of two custom-fabricated Allen keys that serve as precision interface tools for engaging standard optical component knobs. These Allen keys are machined from solid stock with a seamless transition between the shaft and the tool head, forming a single integrated axle. The design was iteratively refined through multiple fabrication cycles, achieving sub–100 μm tolerances. To minimize mechanical resistance and reduce power consumption, the rotational axes are supported by high-performance bearings and shaft collars, ensuring stable alignment and preventing stalling during actuation.

The tool’s software stack uses asynchronous multithreaded control, allowing the Arduino to operate independently of the primary robotic control system. This autonomy enables simultaneous translational and rotational movement, facilitating robust Allen key insertion even in cases of slight misalignment. The insertion routine leverages a helical approach, wherein the tool moves forward while rotating, effectively locating the socket with minimal mechanical stress.

To ensure secure disengagement, the system includes a microbackdriving feature: Upon completing an adjustment, the motors are rotated slightly in the reverse direction of the most recent turn. This subtle motion prevents mechanical binding and ensures smooth retraction of the Allen keys. The combination of precise motor control, low-friction mechanical design, and robust software architecture allows for reliable, repeatable fine adjustments across a variety of optical mounts and experimental configurations with >80% deployment and disengagement accuracy.

### Optical measurements

For the fabrication of the multilayer photonic crystals (measured in Fig. 4D), we performed plasma-enhanced chemical vapor deposition using the SAMCO PD-220 system to deposit a stack of Si and SiO_2_ layers onto a fused silica substrate (microscope coverslips of 180 μm in thickness) with a deposition rate of ∼55 nm/min for both materials. The deposition time was used to control the thicknesses of the individual layers. The photonic crystal consists of eight total layers, alternating between Si and SiO_2_, each with a thickness of 40 nm, except the fifth (Si) layer of 110 nm in thickness, which serves as a defect layer, creating a cavity. The simulated angle- and wavelength-resolved transmission spectrum was calculated using the rigorous coupled wave analysis method, as implemented in Stanford Stratified Structure Solver (S^4^) (*38*). In the simulations, the material model (*n*, *k*) for Si was taken from (*39*) and fit to a fourth-order polynomial in the wavelength range of 400 to 700 nm, while the index of SiO_2_ was assumed to be 1.45.

## Supplementary Materials

### The PDF file includes:

Supplementary Text Figs. S1 and S2 Table S1 Legends for movies S1 to S11 Legends for data S1 to S3
