# Supplementary material for: AI-driven robotics for optics
Source: Sci Adv. 2026 Aug 19;12(34):eaee1381. doi: 10.1126/sciadv.aee1381 (PMC13488923; doi:10.1126/sciadv.aee1381)
Supplement: Supplementary file 1 [file sciadv.aee1381_sm.pdf]

Science Advances
AAAS

Supplementary Materials for

AI-driven robotics for optics

Shiekh Zia Uddin *et al.*

Corresponding author: Shiekh Zia Uddin, suddin@mit.edu; Sachin Vaidya, svaidya1@mit.edu

Sci. Adv. **12**, eaee1381 (2026)
DOI: 10.1126/sciadv.aee1381

The PDF file includes:

Supplementary Text
Figs. S1 and S2
Table S1
Legends for movies S1 to S11
Legends for data S1 to S3

Other Supplementary Material for this manuscript includes the following:

Movies S1 to S11
Data S1 to S3

LLM training curves

The LLM fine-tuning was split into two stages as described in Materials and Methods: *Structure Learning* (Stage 0) and *User Request Understanding* (Stages 1 and 2). The first stage was trained for 349500 iterations. The first session of the second stage was trained for 288000 iterations, and the second session of the second stage was trained for 308000 iterations, with the checkpoint at 282000 iterations was picked based on accuracy evaluation on a validation dataset independently generated from both the training set and the test set. The training curves across stages 0, 1 and 2 are shown in Figure S1.

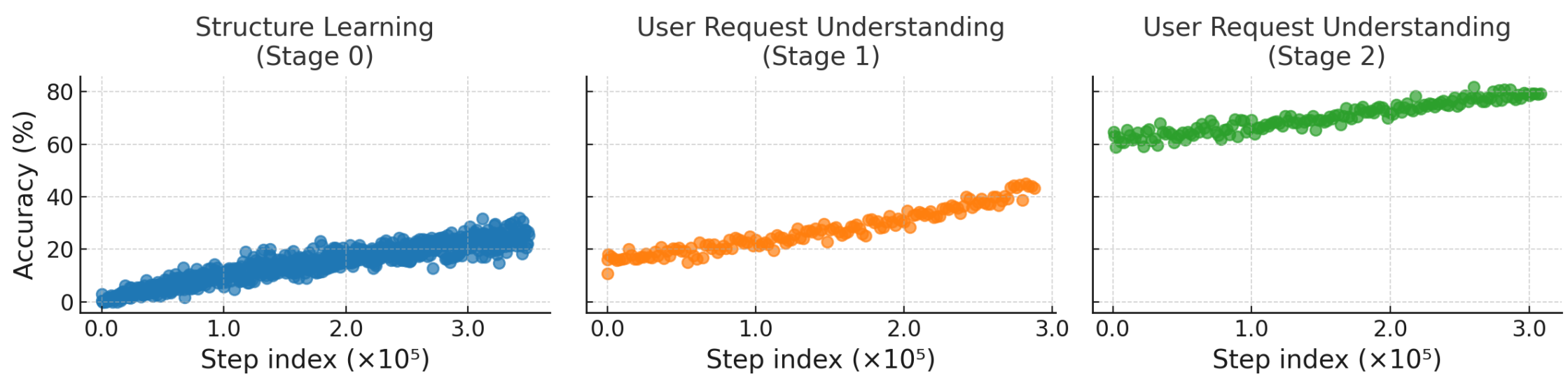

Figure S1: **Training curves for LLM accuracy.** Accuracy (%), defined as the fraction of correct setups over total attempts, of the QuanTA fine-tuned LLaMA model as a function of training step index (scaled in units of 10^5). Each point corresponds to an evaluation checkpoint. The three stages are: structure learning (Stage 0), user request understanding with three requests (Stage 1), user request understanding with random requests (Stage 2).

Robotic arm precision characterization

The robotic arm is a UFACTORY xArm 7 with 7 degrees of freedom, a payload capacity of 3.5 kg, and a reach radius of 61 cm. The manufacturer reports a nominal repeatability of ±0.1 mm; however, under our experimental conditions and measurement pipeline, we determined practical spatial and angular placement precisions of ±0.5 mm and ±0.2°, respectively. These values were obtained by repeatedly commanding the robot to transfer an object between two fixed locations separated by 5 cm, and extracting the resulting distributions of object positions and orientations (Figure S2). The observed scatter plots, which we refer to as pick-and-place repeatability maps, highlight the robot's capacity for consistent handling and placement of objects.

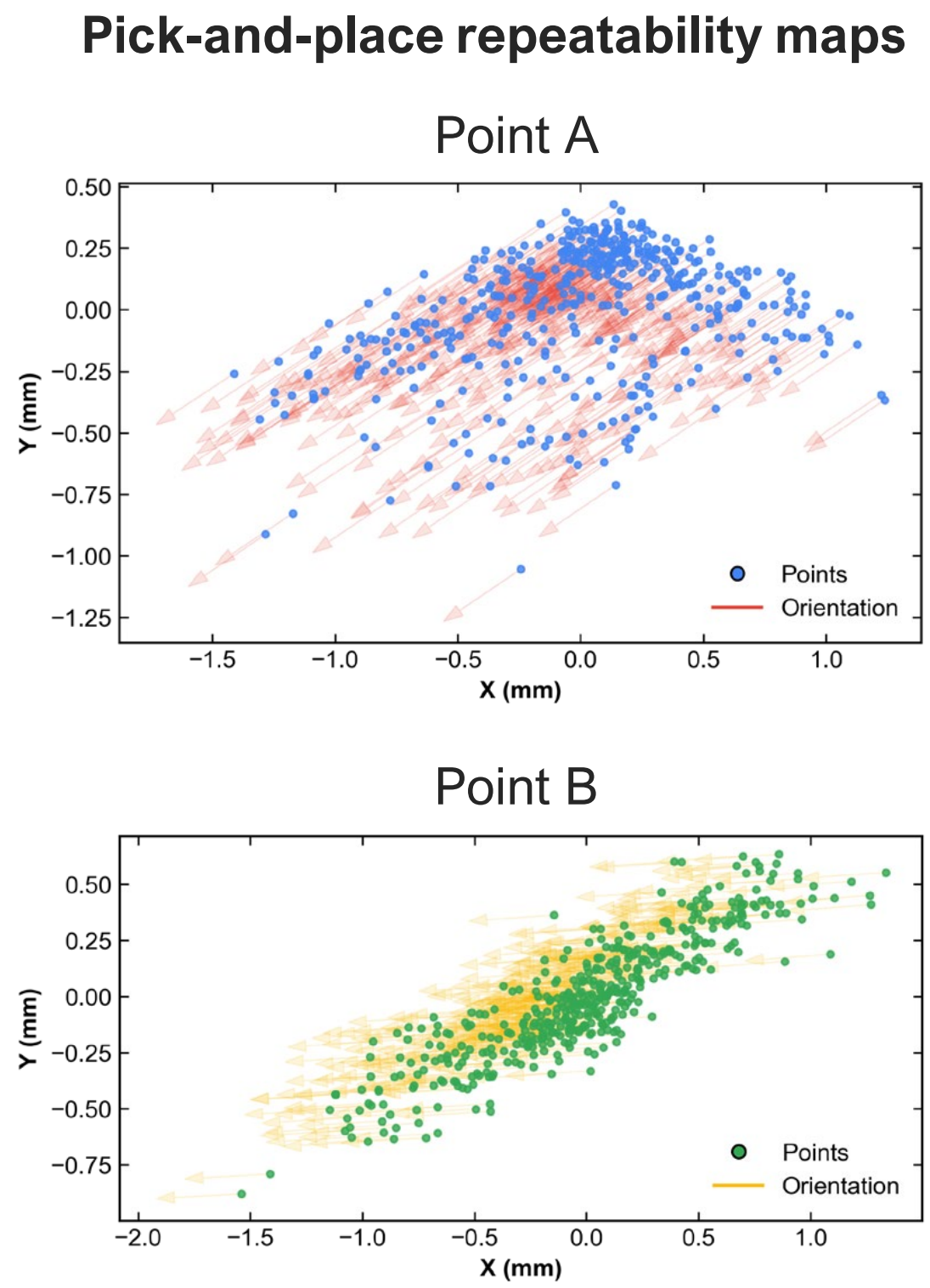

Figure S2: **Pick-and-place repeatability maps.** The robot was programmed to repeatedly transfer an object between two target points (A and B), separated by 5 cm, at a fixed orientation for each point. The scatter of points indicates the final placement positions, while arrows denote the measured orientations at each trial.

Time to completion of optical experiments

Here we report the time to completion of various steps in the optical assembly and measurement tasks. For these tasks the robot motion is deliberately operated at reduced speeds ($\sim$ 5–10$\times$ below hardware limits) to prioritize safety and reliability. In Table S1 below, "Assembly time" reflects static configuration steps; "Alignment time" corresponds to deployment of the fine-alignment tool when required, and "Measurement time" reflects the acquisition time of data and translation/rotation of components. These times were extracted from the corresponding movies. We note that while comparison to human performance is of interest, a rigorous baseline is difficult to establish due to variability in operator expertise, access to specialized tools, and experimental conditions. Nevertheless, the overall times reported below are qualitatively of the same order of magnitude as those achieved by a trained experimentalist.

Task	Assembly time	Alignment time	Measurement time
Interferometer	~ 9 min	~ 5 min	~ 1 min
4f optical system	2 min 55 s	N/A	N/A
Laser direction	N/A	N/A	30 s
M^2 measurement	1 min 10 s	N/A	1 min 15 s
Polarization mapping	2 min	N/A	1 min
PhC spectroscopy	3 min 20 s	N/A	1 min 30 s

Table S1: Summary of assembly, alignment, and measurement times for different optical tasks.

Use of AI-assisted technologies

Large language models (LLMs) were employed solely as copyediting aids in the preparation of this manuscript. In particular, ChatGPT (OpenAI, models GPT-4o and GPT-5) was used to refine sentence clarity, improve grammar, and suggest stylistic edits. The scientific content, experimental design, data analysis, and conclusions were entirely conceived, executed, and validated by the authors. All outputs from the AI tool were carefully reviewed, revised where appropriate, and fully verified by the authors to ensure accuracy and to guard against plagiarism or bias.

Prompts consisted of requests to improve clarity or conciseness of draft sentences or paragraphs (e.g., “Help me check grammar and edit the following text for inclusion in a research paper. Follow a scientific and formal style, appropriate for a research paper, particularly an objective, clear, and technical tone appropriate for a high-quality scientific research article.”).

Movies

The following videos show key capabilities of the robotic system; copy and paste the links to view them.

- Movie S1 - Pick-and-place of optical components:
  `https://youtu.be/aJdHo2FGdSs`
- Movie S2 - Repeated and consistent pick-and-place of components:
  `https://youtu.be/zfZY_MpxlpI`
- Movie S3 - Assembly and teardown of LLM-generated Michelson interferometers:
  `https://youtu.be/bUaSw-KOAX8`
- Movie S4 - Complete assembly and fine alignment of a Michelson interferometer (15× speed):
  `https://www.youtube.com/watch?v=fxjXQAmyJEk`
- Movie S5 - Measurement of beam direction and width:
  `https://youtu.be/nc8Sr8fSde4`
- Movie S6 - Measurement of beam M^2:
  `https://youtu.be/8VmDV0cLOeI`
- Movie S7 - Polarization mapping measurement:
  `https://youtu.be/jFPWFat6xis`
- Movie S8 - Measurement of angle-resolved transmission spectrum of a photonic crystal:
  `https://youtu.be/_PQmDLJ37CA`
- Movie S9 - CAD model of the robot-deployable fine-alignment tool:
  `https://youtu.be/ocdLUdCUIfI`
- Movie S10 - Building a beam cleaning 4f system:
  `https://youtu.be/P7FZhsYfmic`
- Movie S11 - Real-space optimization to create a 2× beam expander:
  `https://youtu.be/3B9kBU7TpOY`

Caption for Movie S1. Pick-and-place of optical components. Video demonstrating computer vision–enabled robotic pick-and-place of optical components, shown from three perspectives.

Caption for Movie S2. Pick-and-place repeatability test. Video demonstrating repeated and consistent transfer of optical component between two fixed points in the working area.

Caption for Movie S3. Assembly and teardown of LLM-generated Michelson interferometers. Video demonstrating robotic assembly of four LLM-generated Michelson interferometers using the following components: two mirrors, one beam splitter, one camera.

Caption for Movie S4. Complete assembly and fine alignment of a Michelson interferometer. Video demonstrating the fully autonomous assembly of a Michelson interferometer followed by the fine-alignment step where the fine-alignment tool is deployed by the robot to align the mirrors (video is played at 15× speed). The result is an interference pattern between the two beams.

Caption for Movie S5. Measurement of beam direction and width. Video demonstrating the autonomous measurement of laser beam direction and width using the robotic arm.

Caption for Movie S6. Measurement of beam M^2. Video demonstrating the autonomous measurement of laser beam M^2 using the robotic arm.

Caption for Movie S7. Polarization mapping measurement. Video demonstrating the autonomous measurement of the spatial variations of beam polarization.

Caption for Movie S8. Measurement of a photonic crystal spectrum. Video demonstrating the autonomous measurement of the angle-resolved transmission spectrum of a multilayer structure (1D photonic crystal). The robot assembles a spectrometer and rotates the photonic crystal in the beam path.

Caption for Movie S9. CAD model of the fine-alignment tool. A video of the CAD model of the robot-deployable fine-alignment tool shown from various angles.

Caption for Movie S10. Building a beam cleaning 4f system. Video demonstrating the autonomous assembly of a beam cleaning 4f optical system.

Caption for Movie S11. Real-space optimization of a beam expander. Video demonstrating the real-space optimization process to expand the input laser beam to a 2× size.

Caption for Data S1. Data for Figure 4(A), (B), (C), (E) of the main text.

Caption for Data S2. Data for Figure 4(D) of the main text.

Caption for Data S3. Data for Figure S2 of the Supplementary Materials.
